# There are more things in heaven and Earth than we dream of in our physiology

**DOI:** 10.1113/JP289962

**Published:** 2025-10-15

**Authors:** Victor A. Maltsev, Michael D. Stern, Edward G. Lakatta

**Affiliations:** ^1^ Intramural Research Program National Institute on Aging, NIH Baltimore Maryland USA

**Keywords:** coupled oscillator, DNA, genetics, pacemaker, physiology, repair mechanisms, sino‐atrial node, small‐world network

In a topical review (Noble, 2026) in this issue of *The Journal of Physiology*, Denis Noble argues that genomics data, for example, genome‐wide association study (GWAS), provide only association with functions or diseases, whereas physiological functions are robust due to redundant mechanisms (e.g. funny current in pacemakers), and that physiology is required to establish genuine causation. He reminds us that information in our large genome would quickly be degraded by replication errors if it were not for active repair mechanisms that correct and preserve. These repair mechanisms are the subject of physiology, without which DNA is merely an inert molecule. Noble seems to express bitter resentment that reliance on genomics has led to closing many physiology departments in the United Kingdom.

If we consider a broader definition of physiology as the study of functions of living organisms and their parts, including the physical and chemical processes enabling life, then both DNA and its repair mechanisms (as a system) belong to physiology. Recognition that most major recent advances in physiology have been achieved synergistically with genetics has led many physiology departments to include molecular biology or genetics, for example, Department of Physiology, Anatomy and Genetics at Oxford University. We prefer this broader view of reality (Fig. 1), in which physiology operates at each level and interfaces to create and maintain life, extending from DNA and its environment to cell, organism, society and their relationships with the planet and the Universe, including numerous feedback circuits and mutual entrainment to maintain the whole. Higher‐level structures confine and direct functions of lower‐level structures, whereas the lower‐level structures micromanage their operations serving the purpose of higher organization to live, survive and prosper.

**Figure 1:**
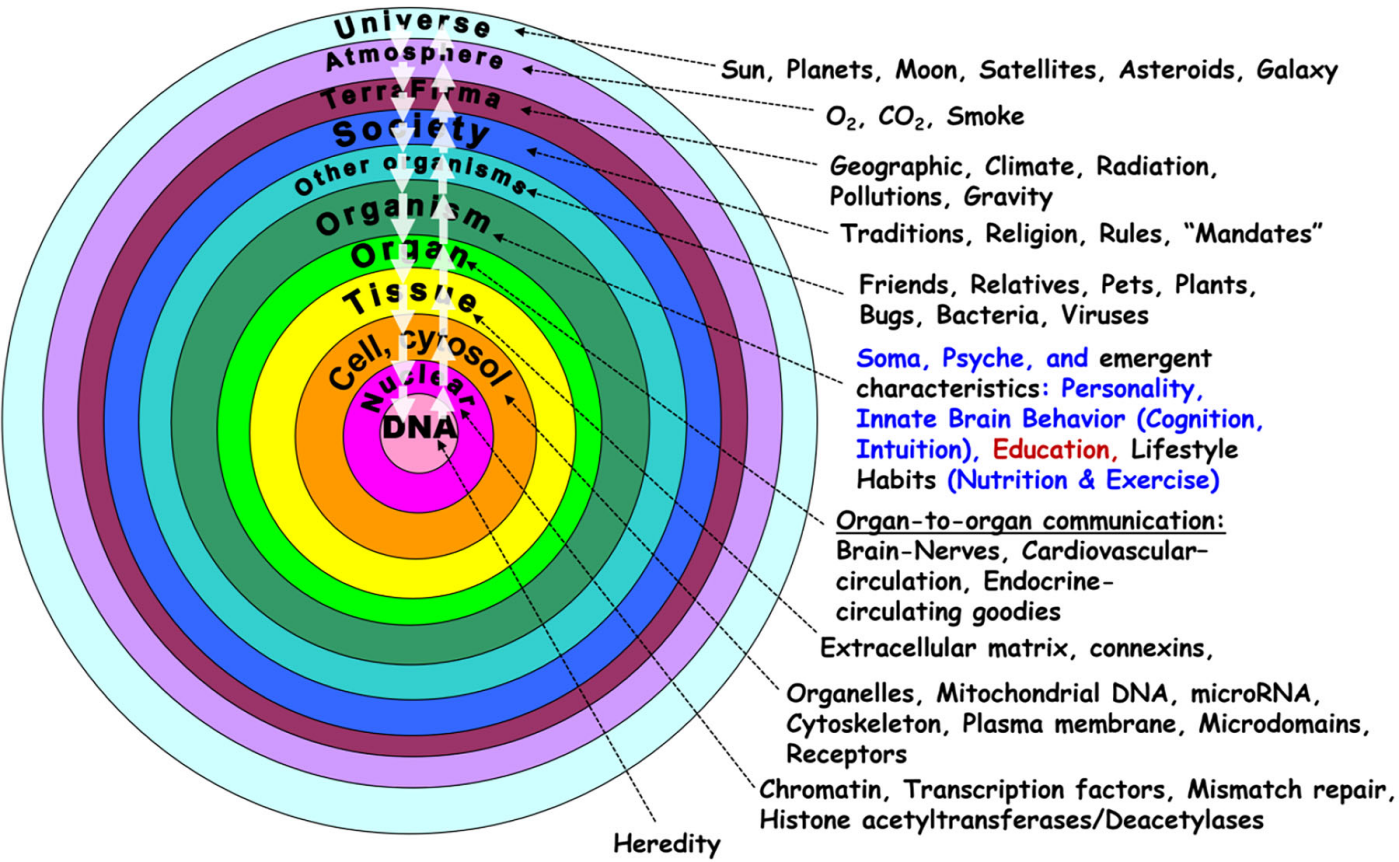
Reality is a system of mutually confined DNA and its environment White arrows designate interactions at interfaces between hierarchy layers. Modified from Lakatta (2015).

For example it is now realized that pacemaker cell function is regulated by a coupled‐oscillator (‘coupled‐clock’) system (Donald & Lakatta, 2023) driven by cAMP produced by neuronal type (AC1, AC8) calcium calmodulin‐activated adenylyl cyclases. When this function is chronically perturbed, a calcium criticality mechanism coupled to a voltage limit‐cycle mechanism within this system also interacts with the DNA to find the best solution to continue functioning. For example AC8 overexpression not only drives the system to fire faster but also changes gene expression (feedbacks) to confine the effects of cAMP (Tarasov et al., 2022). Pacemaker mechanisms are much more complex than previously thought. Sino‐atrial‐node tissue mimics brain cytoarchitecture and function, exhibiting heterogenous local signalling within and among several meshworks of multiple cell types (Donald & Lakatta, 2023). In recent numerical models robust pacemaker function is achieved by a neuronal‐type, small‐world cellular network (Maltsev et al., 2023) and even by a collection of cells that do not fire in isolation (‘dormant cells’) (Maltsev & Stern, 2022). We expect more surprises as we still learn how the pacemaker system operates at the molecular, organelle, cell and tissue levels.

Despite technological revolution and enormous spending on research, we must admit that we only ‘scratched the surface’ of the reality in which living systems operate, repair, regenerate, age and die. The sad truth is that Mother Nature is not the warm and fuzzy protector we would like to believe in. She attacks us on every scale from angstrom‐size thermal vibrations to particles from other galaxies. We maintain our integrity only by virtue of defences at each level that compensate by feedback, repair, redundancies and rebuild from templates. As Feynman famously pointed out, we are not the same person we were a year ago, a month ago or even yesterday; our molecules are constantly recycled. The human is more of an ‘idea’ embodied in a transient collection of molecules. Thus the structure must be continually renewed; otherwise we would quickly disintegrate and diffuse into the cosmos. Only the hierarchy of innumerable defences that require the participation of *both* DNA and its environment protects us from this fate.

But even working together as a physiological system, they eventually begin to miscommunicate and fail. This is what we call ageing and can be generalized as widespread failures to generate, transmit, sense or respond to signals across multiple scales within the system of DNA and its environment and beyond (Fig. 1). Once we reach this triumph of entropy, there is one last‐ditch defence. One cell, protected as much as possible for decades and repeatedly verified, stands to restart the game for a new generation. However as birth rates fall in developed societies, even this last redout is now coming under threat for reasons that are not understood. Although learning the cause may be considered the province of psychology and sociology, it is also a form of ‘social physiology’ on which the survival of our species depends.

## Additional information

### Competing interests

The authors declare no conflict of interest.

### Author contributions

All authors have approved the final version of the manuscript and agree to be accountable for all aspects of the work. All persons designated as authors qualify for authorship, and all those who qualify for authorship are listed.

### Funding

This research was supported by the Intramural Research Program of the National Institutes of Health (NIH). The contributions of the NIH authors are considered Works of the United States Government. The findings and conclusions presented in this paper are those of the authors and do not necessarily reflect the views of the NIH or the U.S. Department of Health and Human Services.

## Supporting information


Peer Review History
