# Supplementary material for: There are more things in heaven and Earth than we dream of in our physiology
Source: J Physiol. 2025 Oct 15;604(13):5084–5. doi: 10.1113/JP289962 (PMC13327752; doi:10.1113/JP289962)
Supplement: Supplementary file 1 — Peer Review History [file TJP-604-5084-s001.pdf]

The Journal of Physiology

There are more things in heaven and Earth than we dream of in our *physiology*

Victor A Maltsev, Michael D Stern, and Edward G Lakatta
DOI: 10.1113/JP289962

Corresponding author(s): Edward Lakatta (lakattae@mail.nih.gov)

The following individual(s) involved in review of this submission have agreed to reveal their identity: Denis Noble (Referee #1)

**Review Timeline:**	Submission Date:	14-Sep-2025
	Accepted:	22-Sep-2025

Senior Editor: Bjorn Knollmann

Reviewing Editor: Axel Loewe

Transaction Report:

(Note: With the exception of the correction of typographical or spelling errors that could be a source of ambiguity, letters and reports are not edited. Depending on transfer agreements, referee reports obtained elsewhere may or may not be included in this compilation. Referee reports are anonymous unless the Referee chooses to sign their reports.)

Dear Dr Lakatta,

Re: JP-P-2025-289962 "**There are more things in heaven and Earth than we dream of in our*physiology***" by Victor A Maltsev, Michael D Stern, and Edward G Lakatta

We are pleased to tell you that your paper has been accepted for publication in The Journal of Physiology.

The last Word (or similar) version of the manuscript provided will be used by the Production Editor to prepare your proof. When this is ready you will receive an email containing a link to Wiley's Online Proofing System. The proof should be thoroughly checked and corrected as promptly as possible.

Authors should note that it is too late at this point to offer corrections prior to proofreading. Major corrections at proof stage, such as changes to figures, will be referred to the Editors for approval before they can be incorporated. Only minor changes, such as to style and consistency, should be made at proof stage. Changes that need to be made after proof stage will usually require a formal correction notice.
All queries at proof stage should be sent to: TJP@wiley.com.

Following copyediting and typesetting the accepted version will be published online.

Yours sincerely,

Bjorn Knollmann
Senior Editor
The Journal of Physiology

IMPORTANT POINTS TO NOTE FOLLOWING YOUR PAPER'S ACCEPTANCE:
If you would like to receive our 'Research Roundup', a monthly newsletter highlighting the cutting-edge research published in The Physiological Society's family of journals (The Journal of Physiology, Experimental Physiology, Physiological Reports, The Journal of Nutritional Physiology, and The Journal of Precision Medicine: Health and Disease), please click this link, fill in your name and email address and select 'Research Roundup':
https://www.physoc.org/journals-and-media/membernews

• TRANSPARENT PEER REVIEW POLICY: To improve the transparency of its peer review process, The Journal of Physiology publishes online as supporting information the peer review history of all articles accepted for publication. Readers will have access to decision letters, including Editors' comments and referee reports, for each version of the manuscript as well as any author responses to peer review comments. Referees can decide whether or not they wish to be named on the peer review history document.

• You can help your research get the attention it deserves! Check out Wiley's free Promotion Guide for best-practice recommendations for promoting your work at: www.wileyauthors.com/eeo/guide. You can learn more about Wiley Editing Services which offers professional video, design, and writing services to create shareable video abstracts, infographics, conference posters, lay summaries, and research news stories for your research at: www.wileyauthors.com/eeo/promotion.

• IMPORTANT NOTICE ABOUT OPEN ACCESS: To assist authors whose funding agencies mandate public access to published research findings sooner than 12 months after publication, The Journal of Physiology allows authors to pay an Open Access (OA) fee to have their papers made freely available immediately on publication.

The Corresponding Author will receive an email from Wiley with details on how to register or log-in to Wiley Authors Services where you will be able to place an order

• You can check if your funder or institution has a Wiley Open Access Account here: https://authorservices.wiley.com/author-resources/Journal-Authors/licensing-and-open-access/open-access/author-compliance-tool.html.

EDITOR COMMENTS

Reviewing Editor:

Thank you for providing this Perspective article, which I consider a valuable contextualisation of the work being referred to.

Please double check the sentence starting with "In recent numerical models, robust pacemaker function is achieved by a neuronal-type, small-world cellular networks..." during the proofing stage for singular/plural correctness.

Senior Editor:

I concur with the reviewing editor. Thank you for you excellent contribution to the Journal!

REFEREE COMMENTS

Referee #1:

Rather than doing a standard Referee report, I have written a short Commentary on the article, attached to this Review site. From this it is clear that I welcome the publication of the article by Maltsev et al.

My Commentary can be used, if the Journal agrees, as a published commentary.

Commentary on Perspective Article:

There are more things in heaven and Earth than we dream of in our *physiology*

Denis Noble
denis.noble@dpag.ox.ac.uk

Departmentof Physiology, Anatomy & Genetics, University of Oxford and Daegu-Gyeongbuk Institute of Science and Technology, South Korea

I welcome the Perspective Article by Maltsev, Stern & Lakatta (2025). It may however be useful to clarify that my article (Noble 2025) should not be interpreted as questioning the importance of *genetics*. I am entirely happy with the name of my own department at Oxford, the department of Physiology, Anatomy & Genetics, while deeply regretting that many other universities in the UK, including very prestigious ones, chose to eliminate the word "physiology" completely.

We needed to know the sequences of DNA that we now call genes, and we certainly need the science of genetics. It is the reliance on genomic sequencing (GWAS) *alone,* as envisaged by those who claim that a “hypothesis is a liability” (Yanai & Lercher, 2020), that is the target of my article. That is what has led to what I call the gene-centric impasse. It is a simple fact that this reliance on associations alone has not led to the many promised benefits in health care, since we cannot then distinguish between association and causation. I therefore agree with the main thrust of the Maltsev, Stern & Lakatta Perspective Article. The Principle of Biological Relativity (Noble, 2012, Figure 4; Noble, 2016, Figure 3.6) is also that there are *two-way* causal interactions between all levels of organisation, as their diagram (Figure 1) also indicates.

Sequencing DNA alone cannot therefore succeed in predicting the development of an individual organism, as neo-Darwinist interpretations of evolutionary biology claim (see reply to Yanai & Lercher by Felin et al, 2021). The related, and more extensive, article by Noble, D & Noble, R (2025) in the journal *Evolutionary Biology* makes this clear.

Figure 2 of that article is similar in intent to Figure 1 in Maltsev, Stern and Lakatta (2025). But an important difference is that the article in *Evolutionary Biology* and the Principle of Biological Relativity make it clear that DNA cannot be the sole source of heredity. We inherit much more than DNA.

Competing interests

There are no competing interests

Author contributions

Denis Noble is the sole author of this work

Funding

No funding supported this work.

Acknowledgements

I thank, Raymond Noble and Reine Bourret for valuable discussion.

References

Felin, T., Koenderink, J., Krueger, J. I., Noble, D. and Ellis, G. F. R. (2021) The data-hypothesis relationship, *Genome Biology*, 22, 57. doi:10.1186/s13059-021-02276-4

Maltsev, V. A., Stern, M.D. & Lakatta, E.G. (2025). There are more things in heaven and earth than we dream of in our *physiology. Journal of Physiology* ***, ***-***

Noble, D. (2012). A theory of biological relativity: No privileged level of causation. *Interface Focus,* 2(1),55–64.

Noble, D. (2016). *Dance to the Tune of Life. Biological Relativity*. Cambridge, CUP.

Noble, D. (2025). The cardiac pacemakers: a paradigm of robustness in evolutionary biology. *Journal of Physiology*. *** ***-*** DOI: 10.1113/JP287139

Noble, D. & Noble, R. 2025. How the Central dogma and the theory of selfish genes misled evolutionary and medical sciences in understanding multi-factorial diseases. *Evolutionary Biology*, 52(2),138–148.

Yanai, I. and Lercher, M. (2020) ‘A hypothesis is a liability’, *Genome Biology*, 21, pp. 231. doi:10.1186/s13059-020-02133-w

Commentary on Perspective Article:

There are more things in heaven and Earth than we dream of in our *physiology*

Denis Noble
denis.noble@dpag.ox.ac.uk

Departmentof Physiology, Anatomy & Genetics, University of Oxford and Daegu-Gyeongbuk Institute of Science and Technology, South Korea

I welcome the Perspective Article by Maltsev, Stern & Lakatta (2025). It may however be useful to clarify that my article (Noble 2025) should not be interpreted as questioning the importance of *genetics*. I am entirely happy with the name of my own department at Oxford, the department of Physiology, Anatomy & Genetics, while deeply regretting that many other universities in the UK, including very prestigious ones, chose to eliminate the word "physiology" completely.

We needed to know the sequences of DNA that we now call genes, and we certainly need the science of genetics. It is the reliance on genomic sequencing (GWAS) *alone,* as envisaged by those who claim that a “hypothesis is a liability” (Yanai & Lercher, 2020), that is the target of my article. That is what has led to what I call the gene-centric impasse. It is a simple fact that this reliance on associations alone has not led to the many promised benefits in health care, since we cannot then distinguish between association and causation. I therefore agree with the main thrust of the Maltsev, Stern & Lakatta Perspective Article. The Principle of Biological Relativity (Noble, 2012, Figure 4; Noble, 2016, Figure 3.6) is also that there are *two-way* causal interactions between all levels of organisation, as their diagram (Figure 1) also indicates.

Sequencing DNA alone cannot therefore succeed in predicting the development of an individual organism, as neo-Darwinist interpretations of evolutionary biology claim (see reply to Yanai & Lercher by Felin et al, 2021). The related, and more extensive, article by Noble, D & Noble, R (2025) in the journal *Evolutionary Biology* makes this clear.

Figure 2 of that article is similar in intent to Figure 1 in Maltsev, Stern and Lakatta (2025). But an important difference is that the article in *Evolutionary Biology* and the Principle of Biological Relativity make it clear that DNA cannot be the sole source of heredity. We inherit much more than DNA.

Competing interests

There are no competing interests

Author contributions

Denis Noble is the sole author of this work

Funding

No funding supported this work.

Acknowledgements

I thank, Raymond Noble and Reine Bourret for valuable discussion.

References

Felin, T., Koenderink, J., Krueger, J. I., Noble, D. and Ellis, G. F. R. (2021) The data-hypothesis relationship, *Genome Biology*, 22, 57. doi:10.1186/s13059-021-02276-4

Maltsev, V. A., Stern, M.D. & Lakatta, E.G. (2025). There are more things in heaven and earth than we dream of in our *physiology*. *Journal of Physiology* ***, ***-***

Noble, D. (2012). A theory of biological relativity: No privileged level of causation. *Interface Focus*, 2(1),55–64.

Noble, D. (2016). *Dance to the Tune of Life. Biological Relativity*. Cambridge, CUP.

Noble, D. (2025). The cardiac pacemakers: a paradigm of robustness in evolutionary biology. *Journal of Physiology*. *** ***-*** DOI: 10.1113/JP287139

Noble, D. & Noble, R. 2025. How the Central dogma and the theory of selfish genes misled evolutionary and medical sciences in understanding multi-factorial diseases. *Evolutionary Biology*, 52(2),138–148.

Yanai, I. and Lercher, M. (2020) ‘A hypothesis is a liability’, *Genome Biology*, 21, pp. 231. doi:10.1186/s13059-020-02133-w
